# Litchi40K v1.0: a cost-effective, flexible, and versatile liquid SNP chip for genetic analysis and digitalization of germplasm resources in litchi

**DOI:** 10.1093/hr/uhaf038

**Published:** 2025-02-10

**Authors:** Lei Zhang, Pengfei Wang, Fang Li, Li Xu, Jietang Zhao, Jingxiao Fu, Jiabin Wang, Hui Zhang, Songang Li, Jiwang Hong, Jian Zheng, Xinping Luo, Huanling Li, Jiabao Wang

**Affiliations:** Institute of Tropical Crop Genetic Resources, Chinese Academy of Tropical Agricultural Sciences, Haikou 571101, China; Institute of Tropical Crop Genetic Resources, Chinese Academy of Tropical Agricultural Sciences, Haikou 571101, China; Environment and Plant Protection Institute, Chinese Academy of Tropical Agricultural Sciences, Haikou 570100, China; Institute of Tropical Crop Genetic Resources, Chinese Academy of Tropical Agricultural Sciences, Haikou 571101, China; College of Horticulture, South China University of Agriculture, Guangzhou 510640, China; Institute of Tropical Crop Genetic Resources, Chinese Academy of Tropical Agricultural Sciences, Haikou 571101, China; Institute of Tropical Crop Genetic Resources, Chinese Academy of Tropical Agricultural Sciences, Haikou 571101, China; Environment and Plant Protection Institute, Chinese Academy of Tropical Agricultural Sciences, Haikou 570100, China; Institute of Tropical Crop Genetic Resources, Chinese Academy of Tropical Agricultural Sciences, Haikou 571101, China; Institute of Tropical Crop Genetic Resources, Chinese Academy of Tropical Agricultural Sciences, Haikou 571101, China; Institute of Tropical and Subtropical Cash Crops, Yunnan Academy of Agricultural Sciences, Baoshan 678000, China; Institute of Tropical and Subtropical Cash Crops, Yunnan Academy of Agricultural Sciences, Baoshan 678000, China; Environment and Plant Protection Institute, Chinese Academy of Tropical Agricultural Sciences, Haikou 570100, China; Institute of Tropical Crop Genetic Resources, Chinese Academy of Tropical Agricultural Sciences, Haikou 571101, China

## Abstract

Genetic breeding and molecular identification in varieties depend on high-performance genotyping tools. The high heterozygosity of the litchi genome contributes to increased resequencing costs and elevated error rates in hybridization-based genotyping methods. In this study, a liquid chip named Litchi40K v1.0 was developed with high-depth resequencing data from 875 litchi samples, and its efficacy was validated across three different populations. In the *L. chinensis* var. *fulvosus* population, three subpopulations characterized by spatial distribution, and a total of 1110 genes were identified in the genomic regions with subpopulation differentiation. Additionally, a total of 30 significant signals associated with diverse agronomic traits were identified. The H002 haplotype of *LITCHI02696*, dominant in the Sub2 subgroup, significantly increased the soluble solid content in the *L. chinensis* var. *fulvosus* population. In a hybrid F_1_ population, a high-density genetic map was constructed and 79 dwarfing-related QTLs were identified with the liquid chip. An NAC transcription factor was identified as a candidate gene with a heterozygous frameshift variant in the male parent. To facilitate the digitization of germplasm resources, 384 SNPs were selected, and the DNA fingerprint map revealed clear genetic relationships and a total of 10 potential synonym groups or instances of bud mutations were identified in 164 main cultivated litchi varieties. This study provides cost-effective, flexible, and versatile liquid chip for genetic analysis and digitalization of germplasm resources in litchi.

## Introduction

Litchi, an evergreen fruit tree from the Sapindaceae family, is a major cash crop in subtropical and tropical regions of China. With a long history of cultivation, China dominates global litchi production, accounting for over 60% of the total planting area and output [1]. However, the industry faces significant challenges, including limited varietal diversity, dependence on a single variety leading to concentrated harvest periods, and severe browning that greatly reduces shelf life, all of which hinder the development of the industry. To ensure the sustainable and high-quality development of the litchi industry, there is an urgent need to develop new varieties with varied ripening times and improved storage resistance.

The rapid advancement of litchi breeding is significantly influenced by the exchange of varieties across different regions. In the early stages of cultivation, the same litchi variety is often given different names depending on the region, while the pattern also observed in other perennial fruit trees like grape [2], apple [3], and pear [4]. These synonyms present considerable challenges the identification of varieties, thereby complicating the selection of appropriate breeding parents. The primary breeding methodologies employed in litchi cultivation include hybrid breeding with known parentage and seedling breeding with ambiguous pollen sources. However, the long juvenile phase of litchi significantly increases breeding costs, so the early screening of hybrid seedlings is crucial to reducing these costs and improving breeding efficiency. Recent research has identified the promoter of the *LcFT1* gene as a useful marker for breeding varieties that flower easily, while the 3781 bp LTR in the *LcCOL307* UTR region may influence flowering and serve as a molecular marker for the development of varieties with varying fruit maturation periods [5–7]. As molecular genetics research in litchi progresses, hundreds of breeding markers linked to various phenotypes will be developed, making high-throughput methods increasingly necessary for their application. Thus, there is an urgent requirement for an affordable, flexible, and accurate high-throughput genotyping tool to facilitate the identification of litchi varieties and early screening of hybrid seedlings.

Resequencing represents a potentially effective approach for litchi breeding, but the high heterozygosity poses a challenge in litchi, requiring deep sequencing to accurately detect heterozygous variants, which consequently increases costs. Enzyme-based genotyping-by-sequencing has successfully generated high-throughput molecular markers in crops and fruits like maize [8], rice [9], and apple [10]. However, the random enzyme sites may not capture the specific breeding markers required for litchi. Solid chips based on sequence hybridization have been successfully applied in species like rice [11], maize [12], and wheat [13], but fluorescent genotyping struggles with heterozygous sites, as evidenced in tea [14]. In contrast, liquid chips offer a more efficient high-throughput genotyping option and have been successfully applied in genetic research and marker-assisted breeding in crops like maize [15], soybean [16], cucumber [17], and watermelon [18], which makes liquid chips a promising solution to address the challenges associated with litchi breeding.

**Figure 1:**
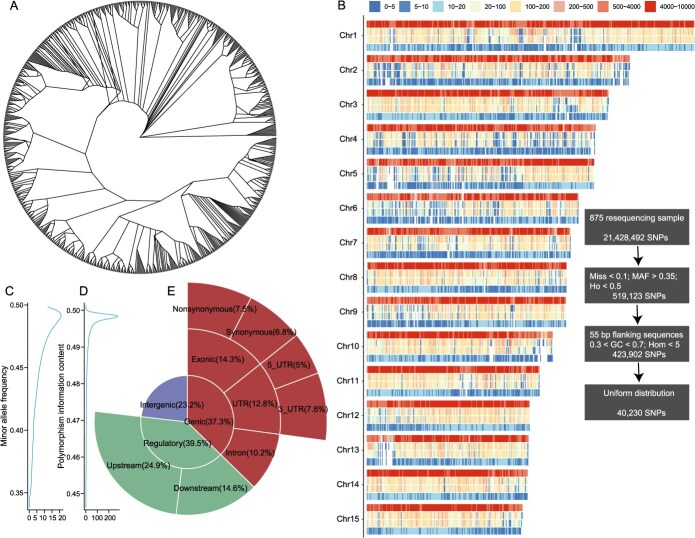
Design and characterization of Litchi40K v1.0. A. The phylogenetic tree of 875 litchi resequencing resources. B. Design process and variation distribution of the Litchi40K v1.0. C. Distribution of the minor allele frequency of the Litchi40K v1.0. D. Distribution of the polymorphic information content of the Litchi40K v1.0. E. The genomic positions of the Litchi40K v1.0.

In this study, we developed Litchi40K v1.0, a liquid chip, using high-depth sequencing data from 875 litchi varieties. We evaluated its performance across multiple applications, including population genetics analysis, association studies, genetic mapping, and pedigree analysis. The evaluation involved 192 *L. chinensis* var. *fulvosus* germplasm resources, 250 F_1_ hybrid individuals, and 164 additional litchi germplasm resources. The affordability, flexibility, and extensive applicability of Litchi40K v1.0 make it a valuable tool for advancing research in litchi genetics and breeding efforts.

## Results

### Design and characterization of Litchi40K v1.0

To ensure broad genetic diversity in the litchi liquid chip, we have assembled the largest litchi resequencing dataset to date. This dataset includes 825 publicly available litchi resources and 50 newly sequenced resources, covering a wide range of geographic regions: 13 varieties from Yunnan, 21 from Sichuan, 145 from Guangxi, 170 from Guangdong, 29 from Fujian, 464 from Hainan, 12 from Southeast Asia, and 2 from Africa. Additionally, the dataset includes 11 *L. chinensis* var. *fulvosus* and 8 *L. chinensis* var. *spontaneous* (Table S1). The broad geographic representation and complex phylogenetic relationships within this collection highlight the rich genetic diversity of the litchi population (Fig. 1A). A total of 21 428 492 high-quality single nucleotide polymorphisms (SNPs) were identified with an average of 45.6 SNPs per kb. By applying several key criteria, including genotyping call rate, minor allele frequency, heterozygosity rate, probe GC content, specificity, and even genome-wide distribution, we developed the liquid chip Litchi40K v1.0, which contains 40 230 high-quality SNPs (Fig. 1B; Table S1).

In the liquid chip Litchi40K v1.0, the average distance between adjacent SNP regions is 11.7 kb, with 90.0% of SNPs concentrated in regions ranging from 0 to 20 kb (Fig. S1). There is a significant positive correlation between the number of SNPs and chromosome length (*R = 0.74, P = 1.8e-3*). The minor allele frequency (MAF) of these SNPs ranges from 0.35 to 0.5, with 68.5% of SNPs having a MAF greater than 0.45. The polymorphic information content (PIC) spans from 0.45 to 0.5, with 80.1% of SNPs showing a PIC value higher than 0.49. Among the identified SNPs, 37.3% are located in genic regions, 39.5% in regulatory regions, and 23.2% in intergenic regions (Fig. 1E). To evaluate the effectiveness of Litchi40K v1.0 in population genetics, we analyzed genetic differentiation using samples from Hainan and Guangdong. The results revealed a three times higher average genetic diversity in Guangdong (8.32e-3 for resequencing, 2.35e-2 for the chip) and Hainan (7.39e-3 for resequencing, 2.08e-2 for the chip), attributed to the higher MAF and PIC value of the selected SNPs in the liquid chip. However, the fixation index (Fst) between the Hainan and Guangdong populations was nearly identical when calculated using both the liquid chip and resequencing data (0.0384 for resequencing, 0.0389 for the chip), demonstrating a strong genome-wide correlation (*R = 0.8*) (Fig. S2).

### Litchi40K v1.0 for population structure analysis of wild populations in litchi

**Figure 2:**
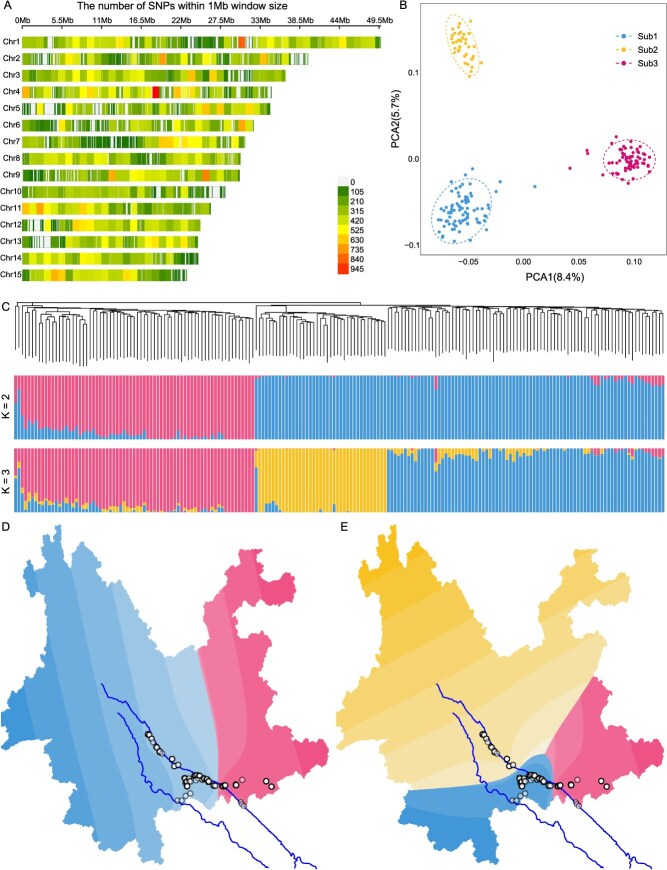
Population structure in the *L. chinensis* var. *fulvosus* population with Litchi40K v1.0. A. The genome-wide SNP identified in the *L. chinensis* var. *fulvosus* population. B. PCA plot of the *L. chinensis* var. *fulvosus* population. C. The phylogenetic analysis and ancestral component analysis from *K* values 2 to 5 of the *L. chinensis* var. *fulvosus* population. D. Spatial population structure with *K* = 2. E. Spatial population structure with *K* = 3.

Population genetic analysis poses a fundamental scientific challenge. To evaluate the performance of the Litchi40K v1.0 liquid chip in this context, we used a publicly available dataset of 192 *fulvosus* germplasm resources, which were genotyped using this chip. In the *fulvosus* population, we identified a total of 149 884 SNPs, with the highest SNP density observed on chromosome 4 at 0.37 SNPs/kb and the lowest on chromosome 10 at 0.26 SNPs/kb (Fig. 2A). Principal component analysis revealed the presence of three distinct subpopulations within the *fulvosus* resources (Fig. 2B). Phylogenetic analysis indicated the presence of two major clades and three minor groups, which were further supported by ancestral component analysis (Fig. 2C; Table S2). Spatial population structure analysis highlighted significant geographical isolation along the Yuanjiang River (Fig. 2D and E). Based on these findings, we classified the 192 *L. chinensis* var. *fulvosus* into three subgroups: Sub1, Sub2, and Sub3.

Among the three subpopulations, Sub2 exhibited the lowest genetic diversity (π = 2.95e-3). Sub3 demonstrated a higher level of genetic diversity with fewer individuals than Sub1, suggesting that genetic diversity within the *L. chinensis* var. *fulvosus* population is influenced by geographic location within the Yuanjiang River. The *Fst* among the subpopulations ranged from 0.21 to 0.27, indicating significant genetic differentiation (Fig. S3A). Similar to genetic diversity, Sub3 exhibited the largest effective population size based on evolutionary history reconstruction (Fig. S3B). A total of 1110 selective genes were identified across the three subpopulations through Fst analysis (Fig. S4A; Table S3). Gene ontology (GO) annotation revealed that these genes were enriched in pathways related to multicellular organismal-level water homeostasis, photosynthetic membrane and miRNA metabolic process. Additionally, 25 homologous genes in litchi were identified, which may play role in flowering, development, and resistance to biotic and abiotic stresses based on functional annotations derived from Arabidopsis (Table S4). Among the identified functional genes, *LITCHI012464*, annotated as a lysine-specific demethylase, shares homologous genes with *AtJMJC* and *PcJMJ25*, which are known to influence flowering and anthocyanin biosynthesis in Arabidopsis and poplar [19, 20]. We identified nine variants within the *LITCHI012464* gene region, which allowed for the classification of the population into three haplotypes (Table S5). Haplotype H001 was the most prevalent in Sub3 (67.2%), while H002 was predominated in Sub2 (89.5%). H003, a hybrid of H001 and H002, had the highest frequency in Sub1 (44.7%) (Fig. S4D). Compared to the Sub2 and Sub3, the Sub1 exhibited higher genetic diversity and Tajima's D values (Fig. S4B). Furthermore, the three haplotypes were significantly associated with solar radiation levels across 12 months (Fig. S4C). These findings suggested that *LcJMJC* may influence the ability of *L. chinensis* var. *fulvosus* to tolerate solar radiation, highlighting its potential as a key target for breeding programs aimed at improving plateau litchi varieties.

### Litchi40K v1.0 for genome-wide association analysis and candidate gene identification in litchi

In addition to population genetic stratification, association mapping between phenotypes and genotypes is a crucial aspect of molecular genetics. We investigated 17 quantitative traits and 24 descriptive traits related to leaves and fruits (Fig. S5). A total of 30 significant signals were identified, associated with 6 quantitative traits and 4 descriptive traits (Table S6; Fig. S6). Notably, three significant signals, Chr1-144 094, Chr2-15 250 996, and Chr5-31 025 202 were linked to multiple complex traits, which simultaneously affect petiole width and petiole area.

**Figure 3:**
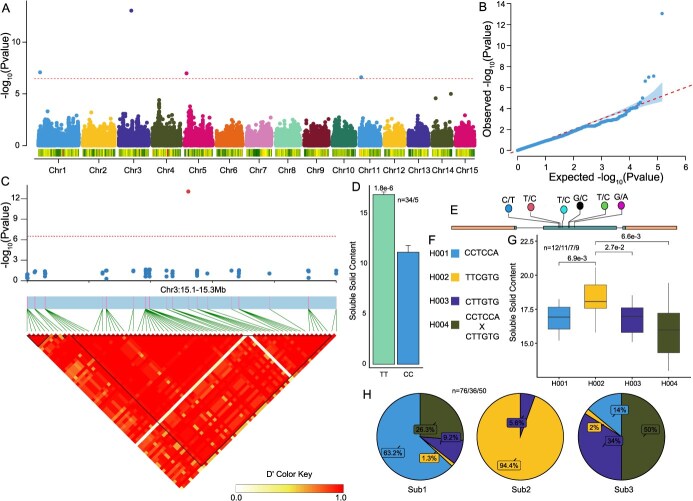
Genome-wide association study in the *L. chinensis* var. *fulvosus* population with Litchi40K v1.0. A. The manhattan plot of soluble solids content. B. The QQ plot of soluble solids content. C. Local manhattan plot and LD block for soluble solids content on chromosome 3. D. Soluble solids content among different alleles at lead SNP. *n* = 34/5 represents the number of different alleles. E. The variants identified in the coding sequence region of LITCHI026966. F. Variant combinations of different haplotypes in LITCHI026966. G. Soluble solids content among different haplotypes. *n* = 12/11/7/9 represents the number of different haplotypes. H. Distribution of LITCHI026966 haplotype in three subpopulations.

Soluble solids content is a key quality trait in litchi with four significant genomic signals identified (Fig. 3A and B). We concentrated on a notable signal located in the central region of chromosome 3, where the lead SNP was found to reduce soluble solids content by 5.7% (Fig. 3D). The confidence interval for this signal is 185 kb and encompasses 8 genes based on linkage disequilibrium, of which, *LITCHI026966*, annotated as glucan endo-1,3-beta-glucosidase, emerged as a candidate gene (Fig. 3C). Six SNPs were detected within the coding sequence of this gene, dividing the *L. chinensis* var. *fulvosus* population into four haplotypes (Fig. 3E, Table S7). H003 appears to be the oldest haplotype, while H002 and H001 derived from H003 through three and four mutations, respectively (Fig. 3F). H004, a hybrid haplotype of H001 and H003, was not found in Sub2, suggesting potential gene flow between Sub1 and Sub3. H002 is predominantly found in the Sub2 (94.4%), but is rare in Sub1 (1.3%) and Sub3 (2%) (Fig. 3H). Significant differences in soluble solids content were observed between H002 and the other haplotypes (Fig. 3G). These findings suggested that H002 haplotype of *LITCHI026966* may be a crucial target for breeding programs aimed at enhancing soluble solids content in litchi.

### Litchi40K v1.0 for genetic mapping of bi-parental population in litchi

**Figure 4:**
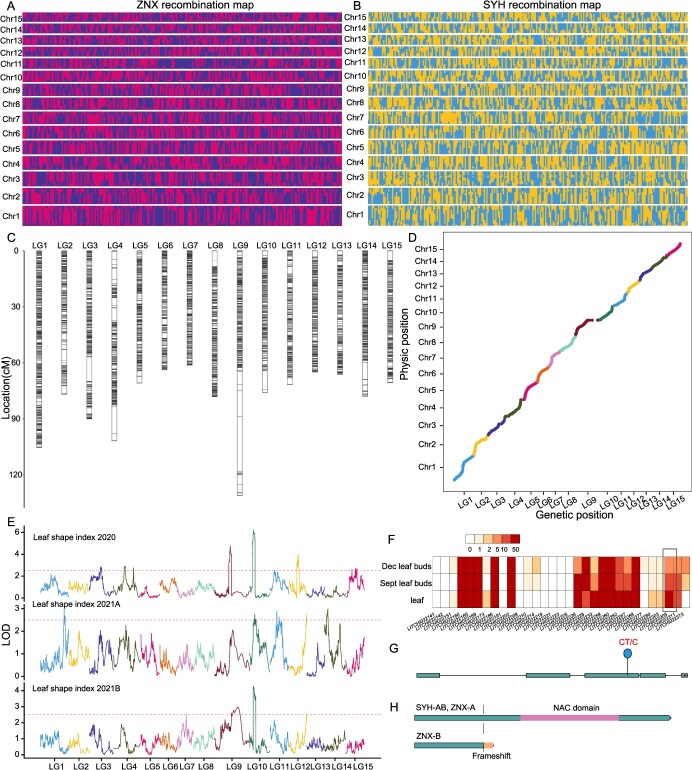
Genetic mapping in the bi-parental population with Litchi40K v1.0. A. The whole genome recombination map of the female founder. B. The whole genome recombination map of the male founder. C. Genetic linkage map of the bi-parental population. D. Collinearity of linkage map and physical map of the bi-parental population. E. The LOD plot of leaf shape index in three years. F. The genes expression of three tissues in confidence interval. G. The frameshift variant in the coding region of LITCHI022305. H. The termination of translation caused by frameshift in ZNX.

Biparental genetic design is a commonly employed methodology for genetic mapping of populations in fruit trees. In this study, we utilized publicly available resequencing data of 264 progeny and their parents, ‘Sanyuehong’ and ‘Ziniangxi’, to simulate and evaluate the performance of the Litchi40K v1.0 liquid chip in genetic mapping for bi-parental population. A total of 267 985 polymorphic SNPs (0/0-0/1, 0/1-0/1, 0/1-1/1) were identified between the parents, with 53.6% of these SNPs exhibited a 0/0-0/1 genotype, indicating greater genetic variation in ‘Ziniangxi’ compared to ‘Sanyuehong’ (Fig. S7). A total of 3440 bins was identified, with bin lengths ranging from 93 bp to 4.9 Mb and an average length of 136 kb. The bin length of recombinant map for ‘Sanyuehong’ range from 117 bp to 6.6 Mb, while for ‘Ziniangxi’, the range extended from 93 bp to 10.2 Mb (Fig. 4A and B). Using recombination data from the F_1_ population, we constructed a comprehensive genetic linkage map spanning 1205.9 cM, with an average genetic distance of 0.35 cM between adjacent markers (Fig. 4C). The collinearity between the genetic linkage map and the physical map was strong (Fig. 4D). Furthermore, recombination rates in the female parent, ‘Sanyuehong’, were significantly higher than that of in the male parent, ‘Ziniangxi’, which was consistent with previous findings in fishes that female gametes exhibit higher recombination rates than male gametes [21] (Fig. S8). The comprehensive analysis revealed 7 segregation distortion regions in the ‘Sanyuehong’ and 10 segregation distortion regions in the ‘Ziniangxi’ (Table S8; Fig. S9).

A total of 79 QTLs associated with seven dwarfing-related traits were identified (Table S9). We focused on a major QTL for the leaf shape index located on chromosome 10, consistently detected across three years. The QTL displayed LOD values ranging from 2.77 to 6.26, accounting for 5.0% to 11.6% of the phenotypic variance, with a confidence interval of 1.6 Mb (Fig. 4E). Notably, the additive effect of the paternal parent was greater than that of the maternal parent, suggesting that the causal variants were originated from ‘Ziniangxi’. Based on gene annotation, heterozygous functional variants in ‘Ziniangxi’ and expression data obtained from the SapBase database, 31 genes were identified (Fig. 4F; Table S10). Among these genes, *LITCHI022305*, annotated as an NAC transcription factor, emerged as a potential candidate gene. The homolog of this gene has been previously reported to play a role in the growth and development in rice [22]. Furthermore, we identified a heterozygous deletion variant at the third exon of ‘Ziniangxi’, resulting in a frameshift that may contribute to the dwarf phenotype (Fig. 4G and H).

### Litchi384 v1.0 for digitalization of germplasm resources in litchi

**Figure 5:**
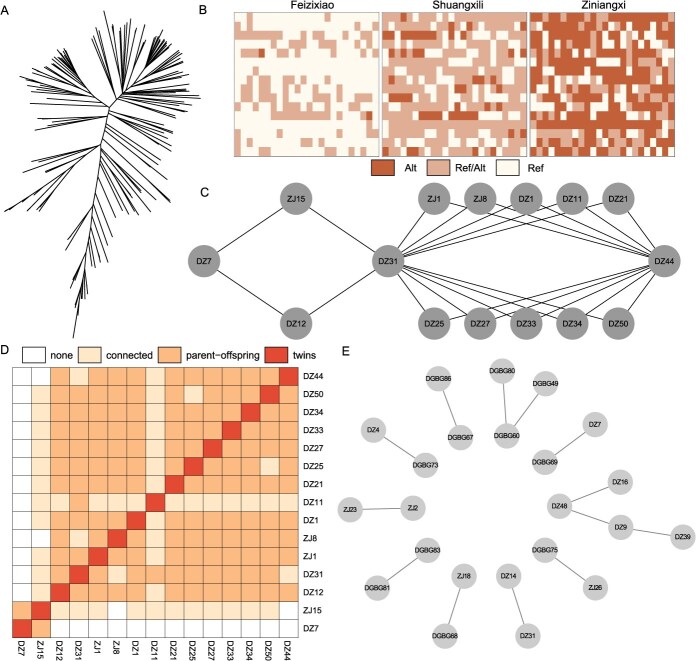
Digitization of germplasm resources in litchi with Litchi384 v1.0. A. The phylogenetic relationships of 164 litchi germplasm resources. B. The QR code of three representative varieties. C. The breeding pedigree of DZ7, DZ31, DZ44, and their offsprings. D. The IBD values of DZ7, DZ31, DZ44, and their offsprings. E. The IBD groups with clone or bud variants relationships.

In addition to molecular genetics research, the liquid chip has proven useful for the digital analysis of germplasm resources. We created the Litchi384 v1.0 fingerprint chip by refining the 40 230 loci of Litchi40K v1.0 to 384 loci (Table S11). A total of 86 litchi germplasm resources from Guangdong and 78 from Hainan were collected, and a comprehensive fingerprint map was constructed with Litchi384 v1.0 (Fig. 5A; Table S12). A QR-code-like three-dimensional SNP markers profile was introduced for each accession (Fig. S10). The QR codes of three typical varieties clearly demonstrated that ‘Shuangxili’ is derived from the hybridization of ‘Feizixiao’ and ‘Ziniangxi’ (Fig. 5B). In the fingerprint map for ‘Feizixiao’, two SNPs exhibited homozygous variants, indicating the presence of somatic cell variant in this sample, which might be caused by long-term clonal propagation.

Using the Litchi384 v1.0, we conducted an identity-by-descent (IBD) analysis on 164 litchi germplasm resources (Fig. S11). DZ31 (‘Ziniangxi’) was utilized as a key representative variety, and it was determined that 33 other resources are found to be closely related to it (Fig. S12; Table S13). Among them, DZ14 was identified as a potential clone or bud mutation of DZ31. Furthermore, 18 resources displayed a parent-offspring relationship with DZ31, while 14 other resources showed also close kinship with it. We verified the kinship relationships among DZ7 (‘Feizixiao’), DZ31, and DZ44 (‘Wuheli’) and their offspring, based on the pedigree information provided by the breeder (Fig. 5C). Most offspring exhibited strong parent-offspring relationships with their respective parents (Fig. 5D). However, DZ12 showed no genomic connection to DZ7, and DZ11 had a low IBD value with DZ44 and other full-sib families. This suggests that DZ12 and DZ11 are descendants of DZ31 but did not originate from pollen of DZ7 and DZ44. Additionally, IBD analysis identified 10 clones or bud variants, which provide valuable genomic insights into the pedigree relationships among different litchi varieties (Fig. 5E; Table S14).

## Discussion

### The Litchi40K v1.0 liquid chip represents a new generation of cost-effective, flexible, and versatile genotyping tool

Compared to traditional resequencing and restriction site-based genotyping, chip-based genotyping presents substantial cost advantages and facilitates the targeted inclusion of specific breeding markers, which are often missed by restriction site approaches [23]. Solid chips, which based on fluorescent hybridization, often have lower accuracy in genotyping heterozygous sites, while liquid chips that use probe-based fragments capture demonstrate genotyping accuracy that is comparable to that of resequencing or restriction site-based methods [14]. Additionally, liquid chips are easier and more affordable to update, as new genotyping sites can be incorporated simply by replacing probes, in contrast to the more expensive updates necessitated by solid chips. Liquid chips also provide higher specificity and accessibility of probes, resulting in deeper sequencing coverage and fewer problems with multiple short-read alignments, thereby further enhancing genotyping precision. Moreover, liquid chips can capture more genetic variants around target sites. In this study, the Litchi4K v1.0 chip identified 149 884 SNPs in 192 *L. chinensis* var. *fulvosus* and 267 985 SNPs between two founders of a hybrid population, exceeding the original design of 40 320 sites.

The liquid chip technology has already been successfully applied in species, such as rapeseed [24], maize [12], and watermelon [18]. To maintain high polymorphic information content and ensure the accuracy of genotyping, low-frequency variant regions were excluded, which might otherwise lead to an overestimation of genetic diversity. Nevertheless, the Litchi40K v1.0 chip demonstrated strong performance in population genetic analyses, association studies, and parental genetic mapping. Notably, three potential candidate genes *LITCHI012464*, *LITCHI026966*, and *LITCHI022305* were identified, which may affect the solar radiation adaptation, soluble solids content and dwarf in litchi.

### The digitalization of germplasm resources using the Litchi40K v1.0 liquid chip aids in litchi variety protection and backbone parent screening

Hybrid and seed breeding are essential methods for advancing litchi breeding. Historically, early records of litchi varieties were disorganized, lacked molecular identification, and were complicated by numerous homonyms across regions [25]. Traditionally, DNA fingerprinting for litchi germplasm resources relied on PCR-based SSR and SNP markers [26, 27]. In this study, DNA fingerprints of 164 germplasm resources were established through automated sequencing, with each assigned a unique molecular QR code. A total of 10 high IBD groups include 23 varieties may represent different names or bud mutations of the same variety. For example, DGBG73 (‘Chenzi’) and DZ4 (‘Dongliu1hao’) from Fujian were molecularly identified as the same variety. Similarly, DZ16 (‘Lingnan39hao’), DZ48 (‘Haikeng11hao’), DZ9 (‘Haikeng22hao’), and DZ39 (‘Haikeng25hao’) are different varieties selected by open pollination in Hainan, but molecular identification showed that they were the same variety. This fingerprinting approach facilitates the digitization of litchi germplasm resources and contributes to the protection and accurate identification of litchi varieties.

Backbone parents are vital for variety improvement and the development of new cultivars. In rice, dwarfing varieties primarily originated from Aizaizhan and Dijiaowujian, while key breeding backbone parents for maize include Huangzaosi and Mo17 [28, 29]. Modern apple varieties are predominantly derived from Fuji, Golden Delicious, Delicious, and Gala in China [30]. Despite the development of numerous litchi varieties, there is no well-defined list of key breeding backbone parents for litchi. Our research suggests that ‘Ziniangxi’, a prominent germplasm resource with large fruit from Hainan Province, is frequently used as a maternal parent in hybrid breeding. Among the 78 germplasms from Hainan sampled in this study, excluding those introduced from other provinces, 42.9% were found to be related to ‘Ziniangxi’, suggesting that ‘Ziniangxi’ may serve as a primary backbone parent for litchi hybrid breeding.

### Early screening of superior alleles using high-throughput genotyping technology represents the future of litchi genetic breeding

Litchi, a tropical evergreen fruit tree, is characterized by a lengthy juvenile period, often requiring more than five years to reach stable flowering. Currently, litchi breeding is predominantly based on traditional hybridization and seed breeding methods. However, the selection of superior plants suitable for actual production is rare, leading to the cultivation of numerous hybrid plants that may never be productive, which results in considerable wastage of resources. To address this issue, early screening of hybrid seedlings is crucial for reducing breeding costs and improving efficiency in litchi breeding. Molecular marker-assisted breeding has been successfully employed in major crops, significantly enhancing breeding efficiency. In litchi, several molecular breeding markers related to flowering have been identified [5–7]. However, traditional PCR verification of these markers is limited in scale, making the detection of a large number of breeding markers associated with various phenotypes challenging. Therefore, high-throughput genotyping methods are needed to overcome these limitations. The Litchi40K v1.0 liquid chip presents a flexible and cost-effective alternative to traditional solid-phase chips, which are costly to update and iterate. The liquid-phase chip can be easily updated to accommodate new molecular markers as advancements in molecular genetics are made, thereby facilitating for efficient high-throughput typing and timely adjustments in breeding programs. Whole-genome selection breeding has been effective in rice [31] and dairy cattle [32], though it has yet to be reported in litchi. The affordable Litchi40K v1.0 liquid-phase chip provides stable genotyping results that could facilitate whole-genome selection breeding in litchi. The integration of early screening with molecular marker assistance and whole-genome selection using the Litchi40K v1.0 chip represents a promising advancement for litchi genetic breeding.

## Materials and methods

### Plant materials and genome resequencing

The 50 litchi resources for resequencing were obtained from the filed gene bank of litchi located in Danzhou, Hainan. The 164 litchi resources of DNA fingerprint came from the filed gene bank of litchi located at Hainan, Zhanjiang, and Dongguan Botanical Garden. Total DNA was extracted from fresh leaves of each accession using the modified CTAB method. Two DNA libraries were constructed using the NGS pipeline and the special fragment capture pipeline, respectively (Table S15 , S16). Then paired-end sequencing was performed on the Nova 6000 platform in Molbreeding (Sanya, China) with a read length of 150 bp. The sequencing data of samples for resequencing and chip was 16 and 2.5 Gb, respectively.

### Genome-wide representative SNP identification

825 resequencing data with an average depth greater than 40X were obtained from PRJNA765302. Low-quality reads and sequences from the raw resequencing reads of 875 samples were removed and trimmed by Fastp software [33]. Then, paired-reads were aligned to the litchi reference genome (Feizixiao, http://www.sapindaceae.com) with the MEM algorithm of BWA [34]. Samtools [35] was used to sort and compress alignment files and PCR duplicates were removed by MarkDuplicates module in Picard software (https://broadinstitute.github.io/picard/). SNPs were identified by the HaplotypeCaller and GenotypeGVCFs program with GVCF mode in the Genome Analysis Toolkit [36], while the SNP filtering criterions were ‘QD < 2.0’ or ‘SOR > 4.0’ or ‘FS > 60’ or ‘MQ < 40.0’ or ‘MQRankSum < −12.5’ or ‘ReadPosRanSum < −8.0’.

Representative SNPs were obtained through a comprehensive methodology. The criteria for inclusion were as follows: 1, missing rate < 0.1, minor allele frequency > 0.35 and observed heterozygosity <0.5 using plink; 2, the GC content of 110 bp flanking sequence ranged from 0.3 to 0.7 and there were less than 5 homologous alignment regions (more than 40 bp of identical sequences, more than 85% similarity over 80 bp, or more than 95% similarity over 70 bp) in the 110 bp flanking sequence; 3, conform to the standard of uniform distribution with a window of 100 kb.

### SNP identification for *L. chinensis* var. *fulvosus*, DNA fingerprint and bi-parental populations

About, 192 samples of *L. chinensis* var. *fulvosus* population were download from PRJCA026922. Totally, 264 samples of bi-parental population were download from PRJCA021038. The raw reads were trimmed and clipped the high-quality part of read by Fastp software. Subsequently, BWA was used to align the clean reads to the reference genome and Samtools was used to sort and convert the SAM file to BAM file. The MarkDuplicates module in the Picard software was used to remove the PCR duplicates generated during library construction. The HaplotypeCaller and GenotypeGVCFs program in the Genome Analysis Toolkit were used to identify SNPs with GVCF mode. The hard filtering conditions of GATK were ‘QD < 2.0 || SOR > 4.0 || FS > 60 || MQ < 40.0 || MQRankSum < −12.5 || ReadPosRanSum < −8.0’. SNPs with missing rates below 20%, minor allele frequency above 5% and located in the capture regions were kept for downstream analysis.

### Population genetics analysis

Independent SNPs were extracted using the independent pairwise method with a linkage disequilibrium threshold of 0.2 in plink [37]. The distance matrixes were calculated in plink, and phylogenetic trees were constructed by MEGA software [38], while R/ggtree package was used to display the trees [39]. Principal component analysis was performed with independent SNPs in plink. The maximum-likelihood clustering program ADMIXTURE [40] was used to estimate individual ancestry, while CLUMPP software [41] was used to permute the cluster output from ADMIXTURE. The R/tess3r package was used to estimate population genetic structure on geographic maps [42]. The IBD analysis of 164 resources was performed in plink.

### Genetic diversity and population difference inference

Genetic diversity(π) of the resequencing data was calculated in 100 kb windows and 10 kb step size by VCFtools [43]. Genetic diversity(π) of the chip data was calculated using python module tajimas-d (https://github.com/not-a-feature/tajimas_d) across all fragments. The whole genome population differentiation coefficients (*Fst*) were calculated using VCFtools in 100 kb windows and 10 kb step size. Starway Plot [44] was used to infer historical changes in the effective population size (*Ne*) of different subgroups using default parameters with the entire genomic dataset, while assuming a generation time of 30 years in *L. chinensis* var. *fulvosus* and a mutation rate of 7.7e^−9^ mutations per generation [7]. Genes located within these selective regions underwent a functional enrichment analysis based on GO by R/clusterProfiler [45].

### Genome-wide association analysis

Genome-wide association analysis was conducted using the FarmCPU model, which is implemented in the package R/rMVP [46]. The analysis included the incorporation of the first five principal components as covariates and the use of kinship to control for population structure. A strict Bonferroni correction was used to set the threshold value for GWAS.

### Bi-parental linkage mapping analysis

Only SNPs with diallelic validity between the parents were retained, while the genotype of either parent was heterozygous. All skewed SNPs were removed from the population and a neutral genetic linkage map was constructed in the Lepmap3 software [47]. SNPs were converted to BIN type based on genetic map information. Genetic analysis was performed in GACD software with ICIM model [48]. LOD threshold was set to 2.5.

## Supplementary Material

Web_Material_uhaf038

## Data Availability

All raw reads generated for the individuals in the study have been deposited in the National Genomics Data Center under BioProject PRJCA029560 and PRJCA029562.
