# Supplementary material for: Litchi40K v1.0: a cost-effective, flexible, and versatile liquid SNP chip for genetic analysis and digitalization of germplasm resources in litchi
Source: Hortic Res. 2025 Feb 10;12(5):uhaf038. doi: 10.1093/hr/uhaf038 (PMC11997437; doi:10.1093/hr/uhaf038)
Supplement: Web_Material_uhaf038 [file web_material_uhaf038.zip › supFig.docx]

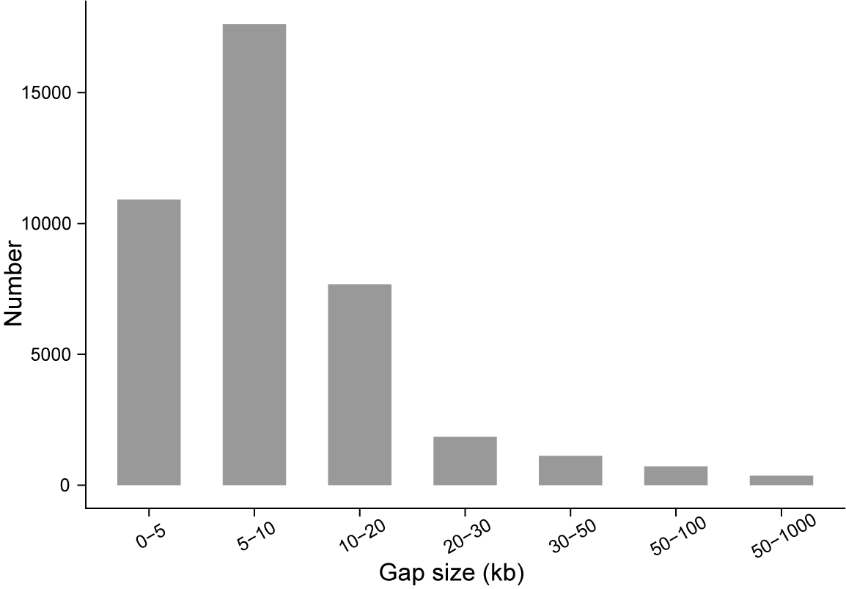


**Supplementary Fig. 1 The number of gap size between two adjacent SNPs.**


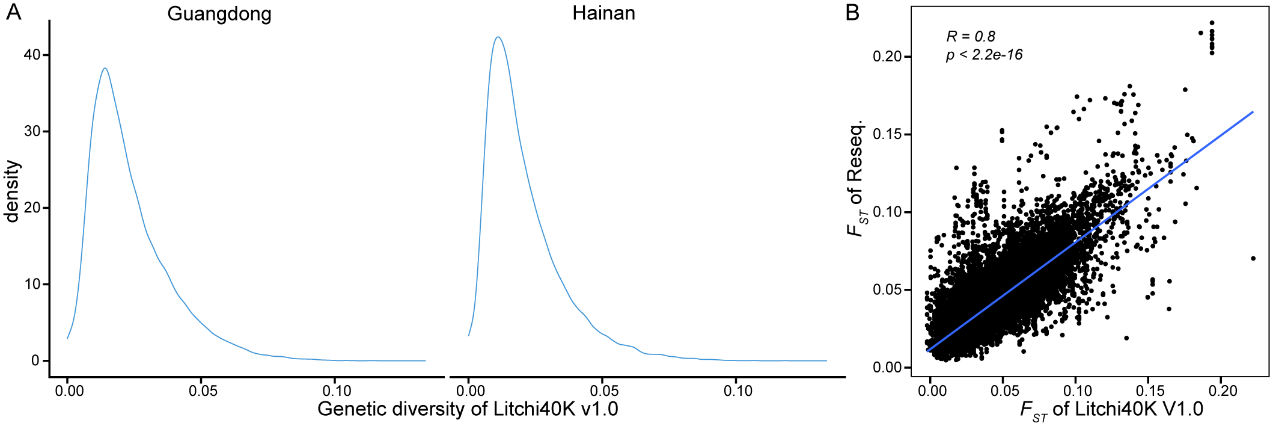


**Supplementary Fig. 2 Characteristics of the Litchi40K v1.0 in population genetics**

**A.** The distribution of genetic diversity in two groups. **B**. Correlation analysis of Fst value by resequencing and Litchi40K v1.0.


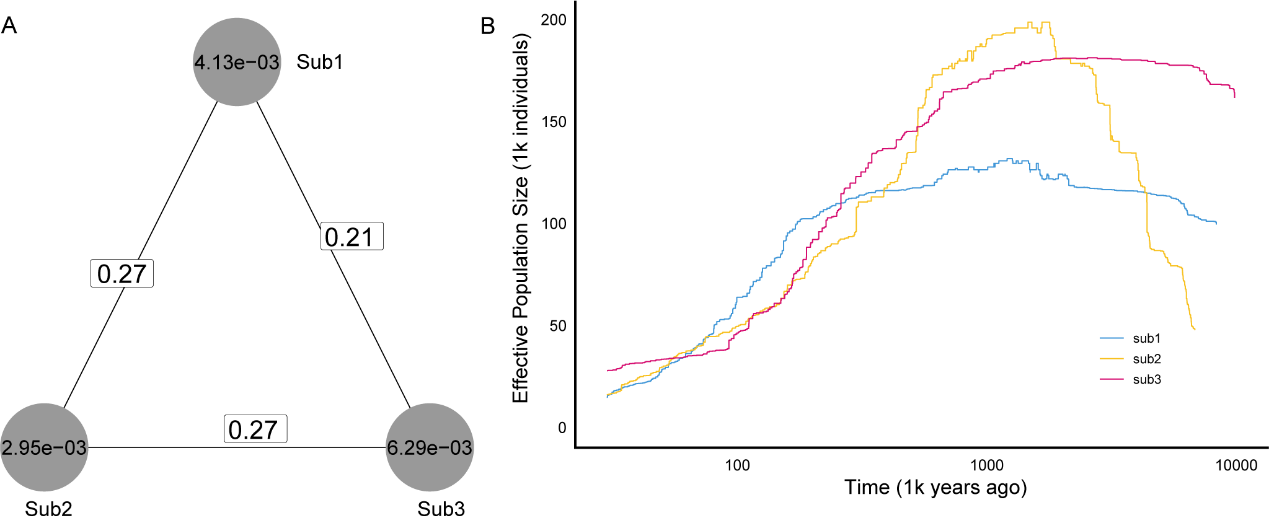


**Supplementary Fig. 3 Genetic characteristics of the *L. chinensis* var. *fulvosus* population**

**A.** Genetic diversity and Fst values of the three subpopulations. **B**. Effective population size of the three subpopulations.


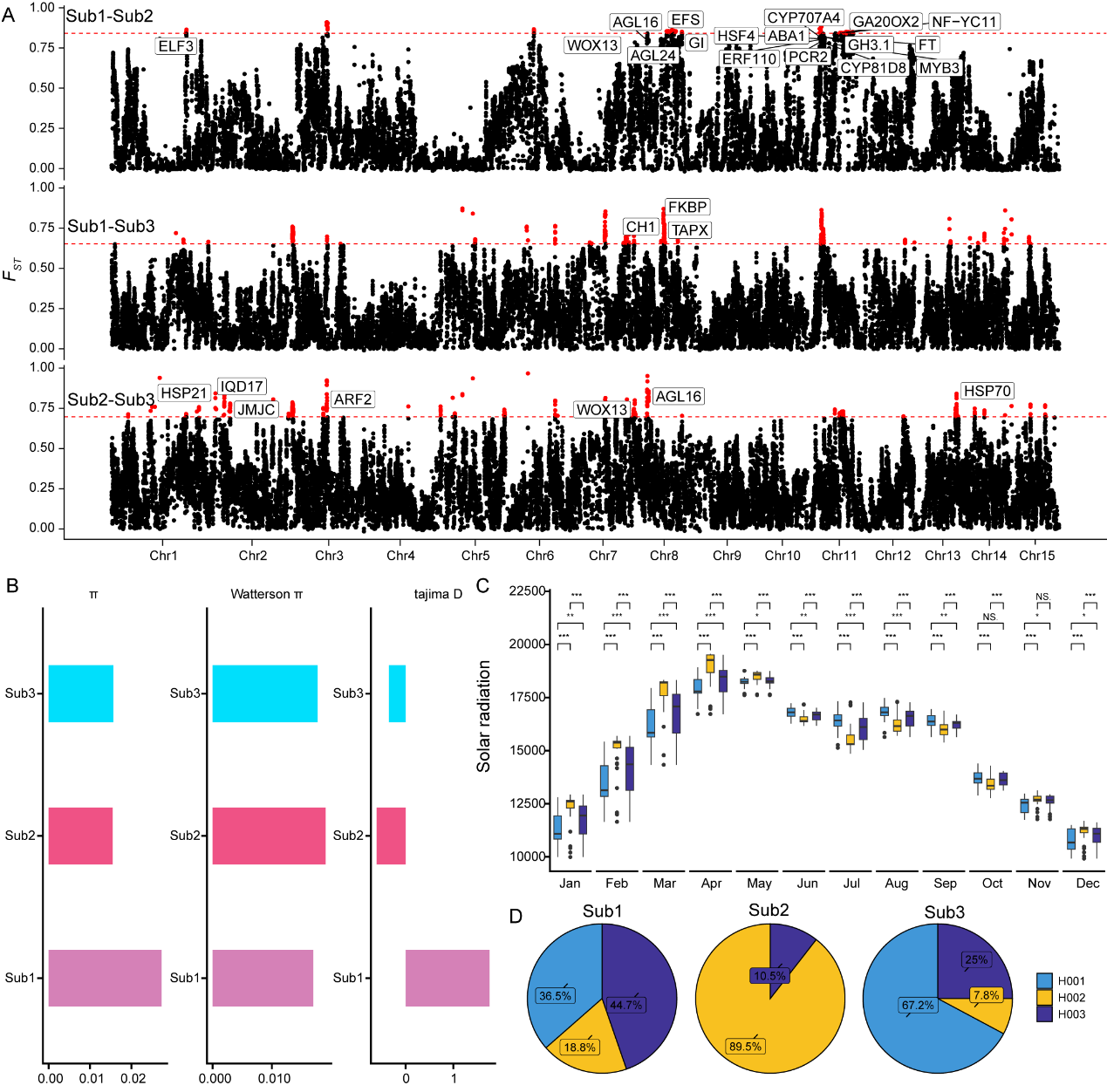


**Supplementary Fig. 4 Selective signals in the *L. chinensis* var. *fulvosus* population**

A. Genetic differentiation among the three subpopulations. B. Genetic parameters of the LITCHI012464 region in different subpopulations. C. Solar radiation in 12 months among different haplotypes. D. Distribution of LITCHI012464 haplotype in different subpopulations.


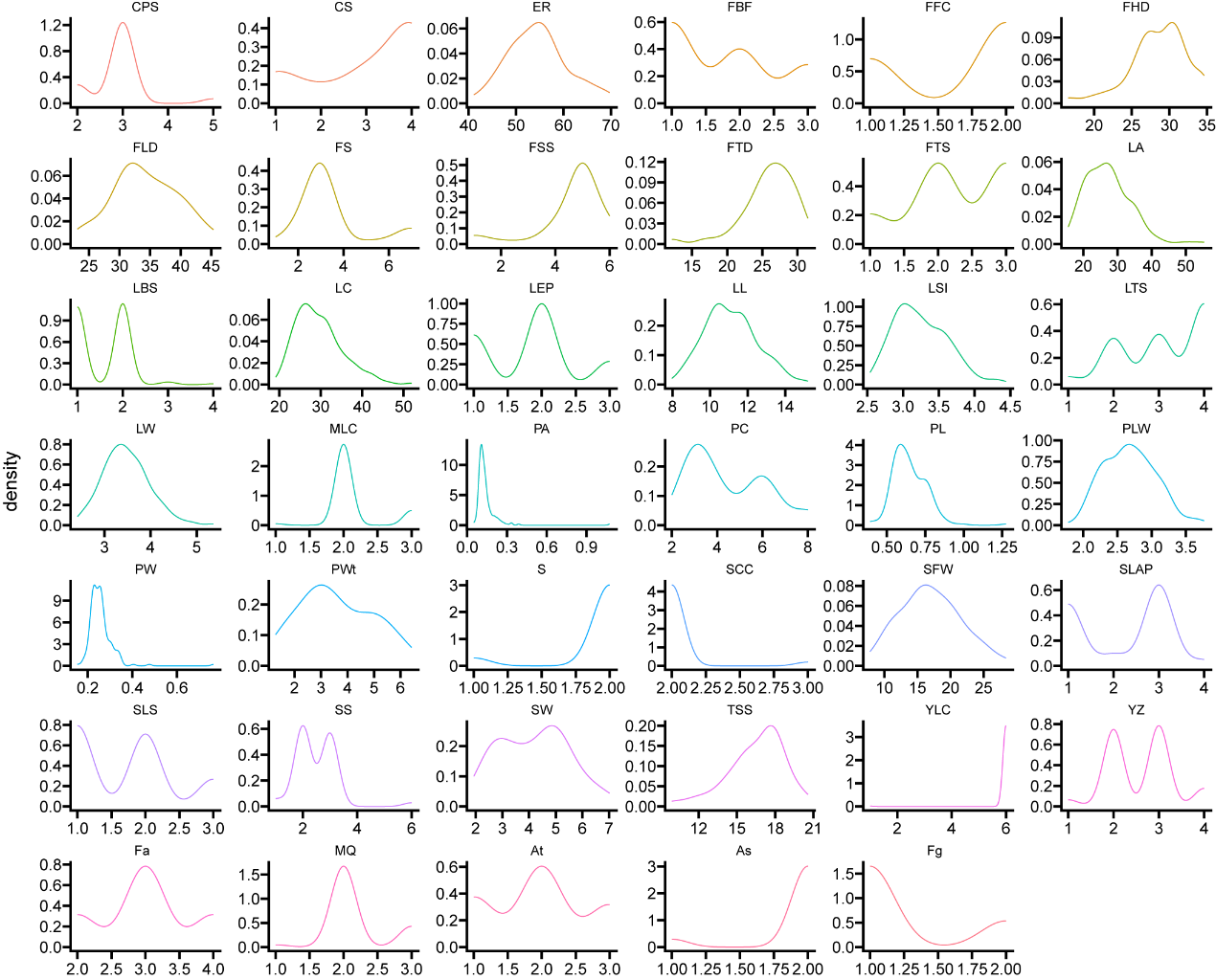


**Supplementary Fig. 5 Distribution of 41 traits in the *L. chinensis* var. *fulvosus* population**


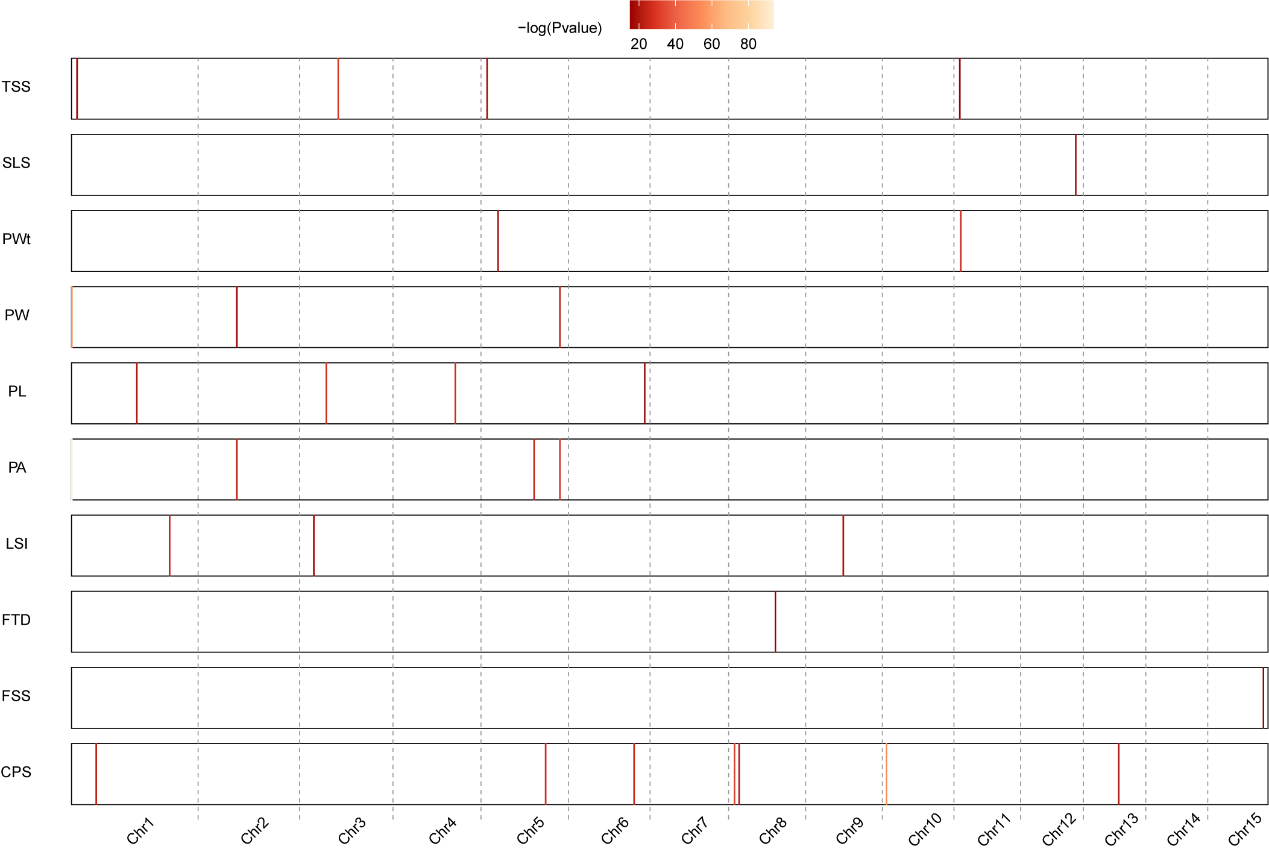


**Supplementary Fig. 6 Significant signals of 10 traits in the *L. chinensis* var. *fulvosus* population**


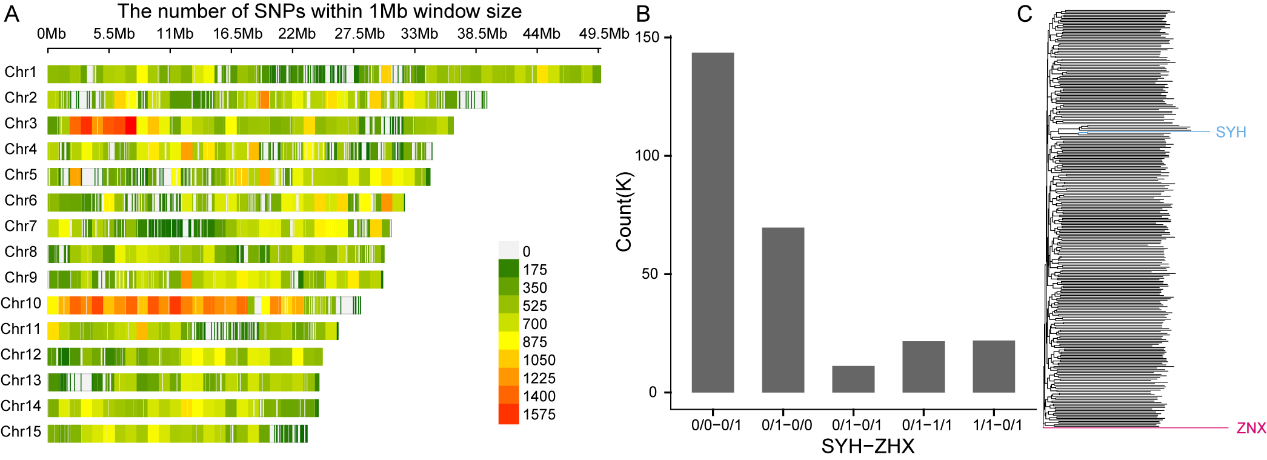


**Supplementary Fig. 7 Characteristics of the bi-parental population**

**A**. Distribution of genetic variant between two parents. **B**. The number of different types of genetic variant between two parents. **C**. The phylogenetic tree of the bi-parental population.


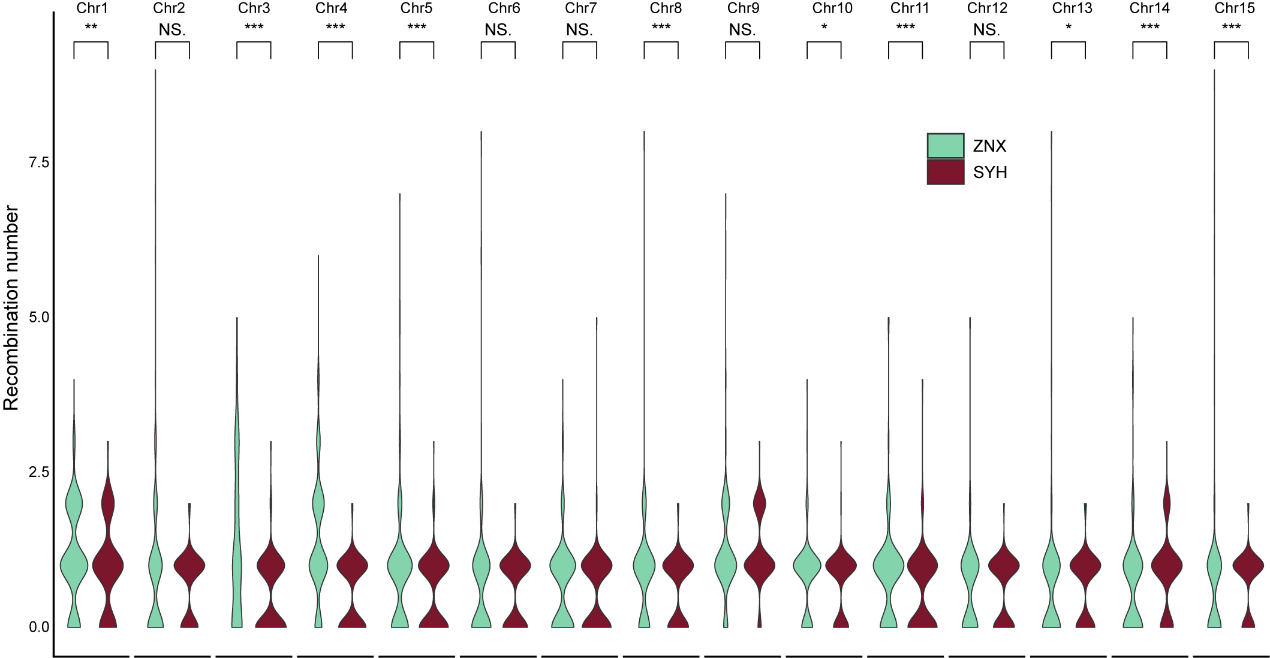


**Supplementary Fig. 8 Comparison of the number of recombinants on the whole genome between the two parents**


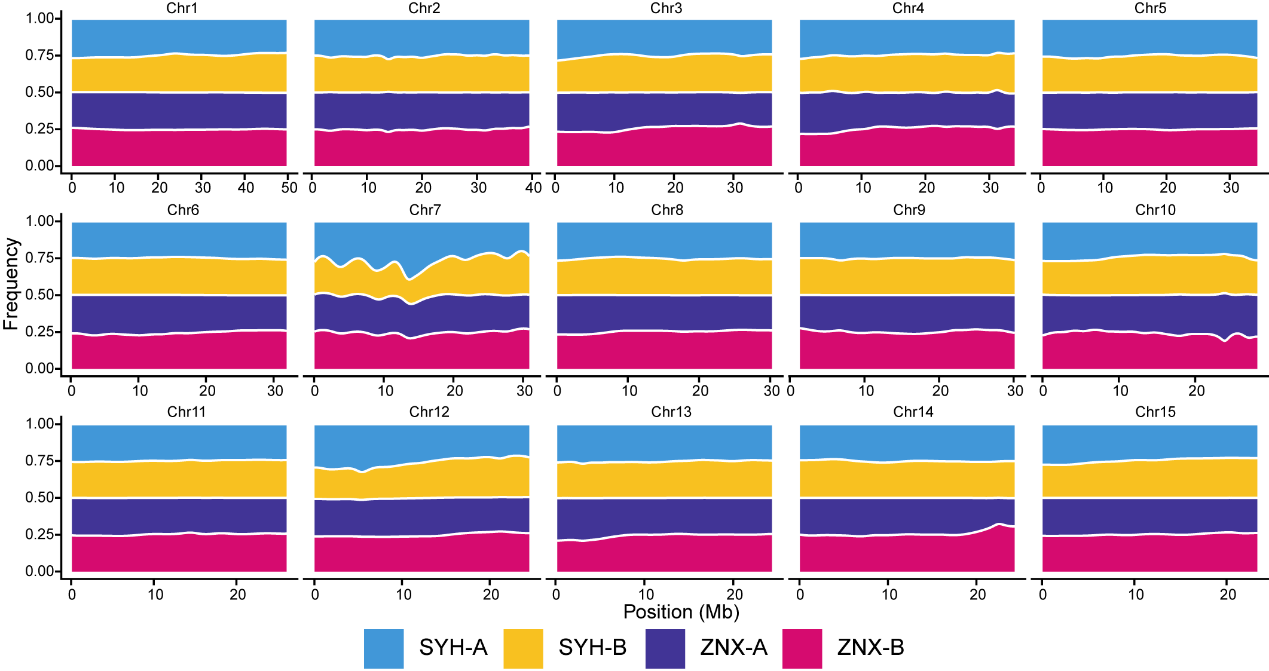


**Supplementary Fig. 9 Genetic distribution of the two parents in the offspring population**


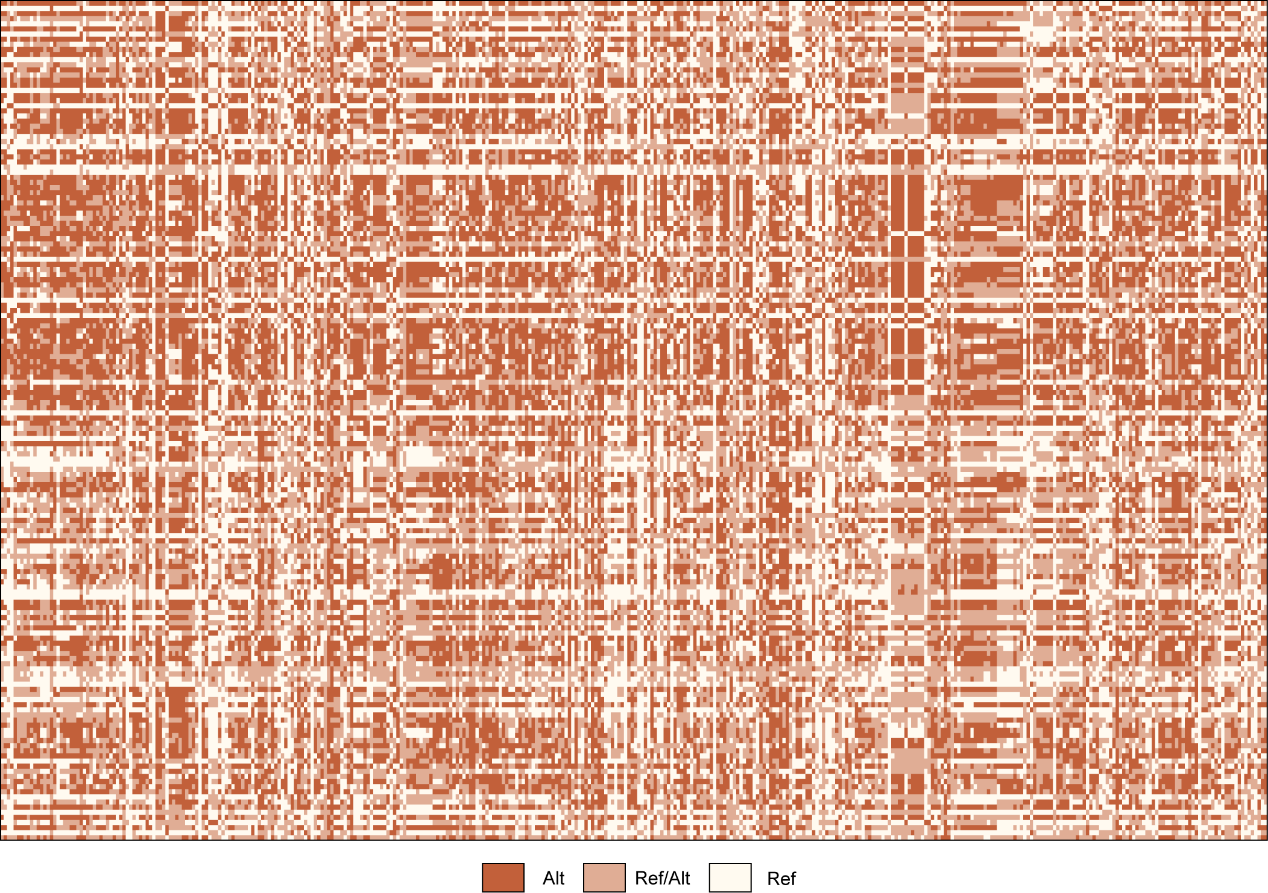


**Supplementary Fig. 10 The QR code for 164 germplasm resources in litchi**


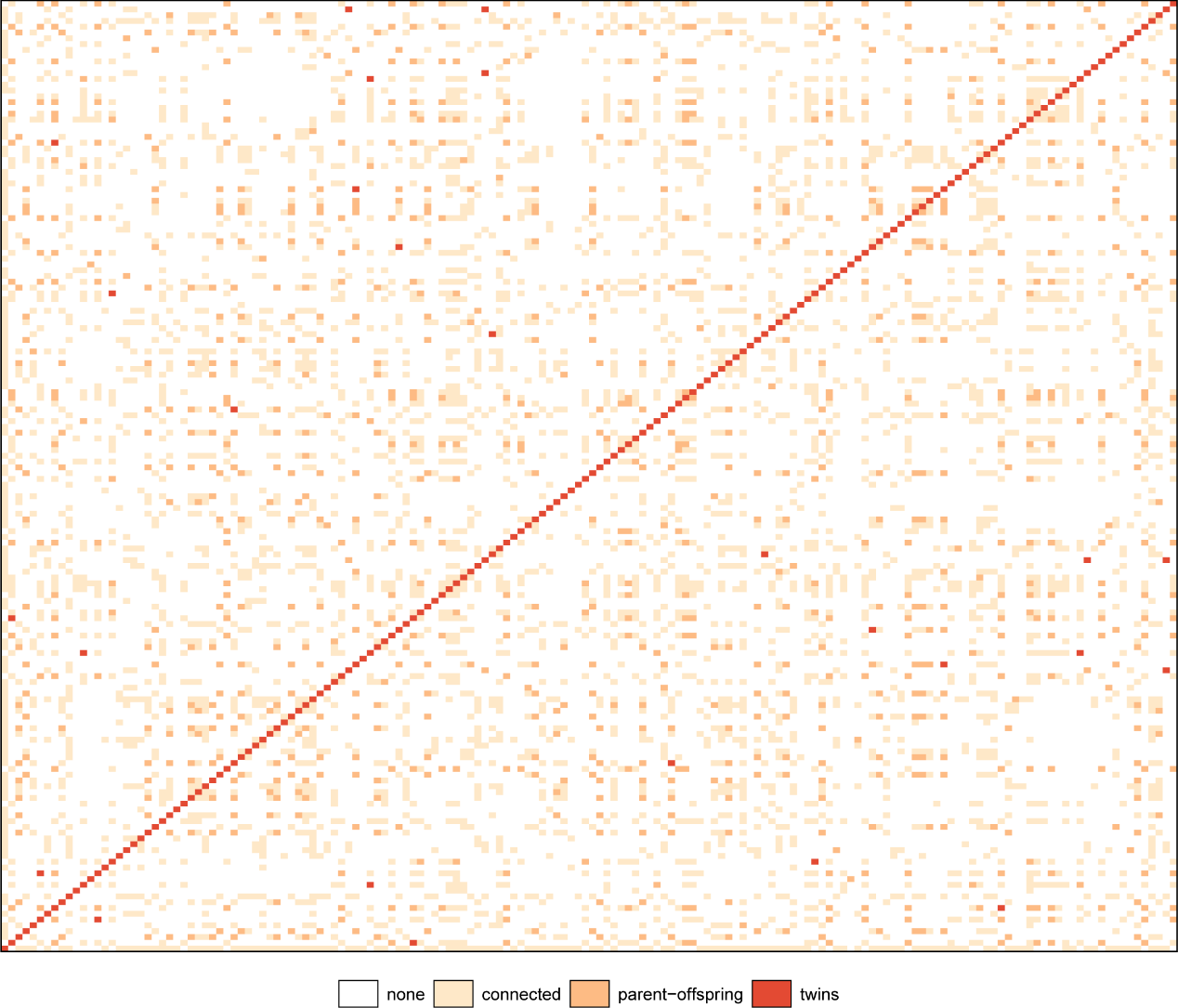


**Supplementary Fig. 11 The kinship relationships of the 164 germplasm resources in litchi**


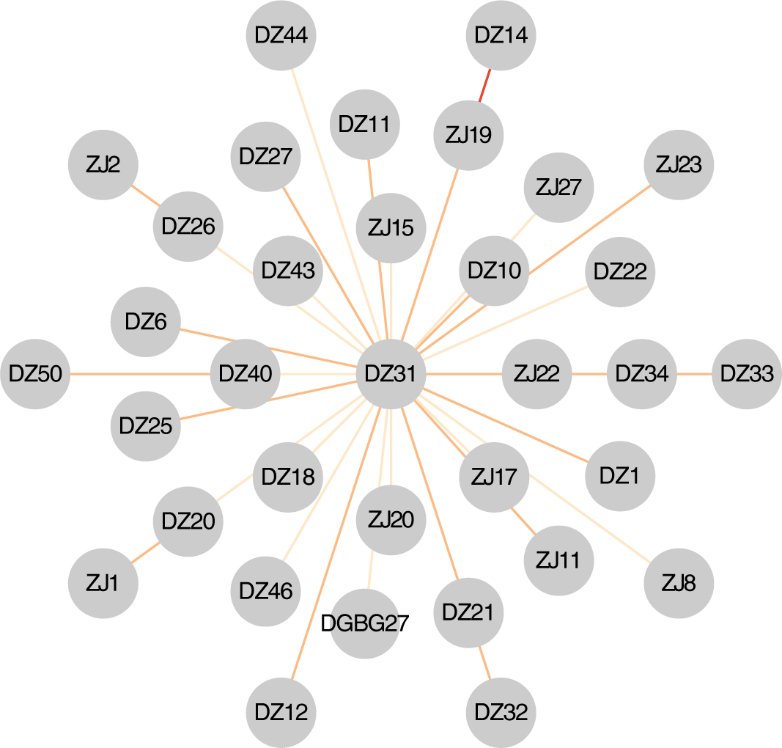


**Supplementary Fig. 12 The kinship relationships of DZ31**
